# Biophysical Assessment and Predicted Thermophysiologic Effects of Body Armor

**DOI:** 10.1371/journal.pone.0132698

**Published:** 2015-07-22

**Authors:** Adam W. Potter, Julio A. Gonzalez, Anthony J. Karis, Xiaojiang Xu

**Affiliations:** Biophysics and Biomedical Modeling Division, United States Army Research Institute of Environmental Medicine, Natick, Massachusetts, United States of America; Michigan State University, UNITED STATES

## Abstract

**Introduction:**

Military personnel are often required to wear ballistic protection in order to defend against enemies. However, this added protection increases mass carried and imposes additional thermal burden on the individual. Body armor (BA) is known to reduce combat casualties, but the effects of BA mass and insulation on the physical performance of soldiers are less well documented. Until recently, the emphasis has been increasing personal protection, with little consideration of the adverse impacts on human performance.

**Objective:**

The purpose of this work was to use sweating thermal manikin and mathematical modeling techniques to quantify the tradeoff between increased BA protection, the accompanying mass, and thermal effects on human performance.

**Methods:**

Using a sweating thermal manikin, total insulation (I_T_, clo) and vapor permeability indexes (i_m_) were measured for a baseline clothing ensemble with and without one of seven increasingly protective U.S. Army BA configurations. Using mathematical modeling, predictions were made of thermal impact on humans wearing each configuration while working in hot/dry (desert), hot/humid (jungle), and temperate environmental conditions.

**Results:**

In nearly still air (0.4 m/s), IT ranged from 1.57 to 1.63 clo and i_m_ from 0.35 to 0.42 for the seven BA conditions, compared to I_T_ and i_m_ values of 1.37 clo and 0.45 respectively, for the baseline condition (no BA).

**Conclusion:**

Biophysical assessments and predictive modeling show a quantifiable relationship exists among increased protection and increased thermal burden and decreased work capacity. This approach enables quantitative analysis of the tradeoffs between ballistic protection, thermal-work strain, and physical work performance.

## Introduction

Military personnel commonly work at moderate-to-high intensities for prolonged periods in dangerous combat environments under harsh and variable environmental conditions [1]. In order to defend against the elements and enemies, individual soldiers wear protective clothing, body armor (BA), and helmets. In addition to their basic uniform, typical dismounted soldier’s ensembles include BA in the form of hard ceramic plates, and soft armor (e.g., Kevlar).

In order to tailor BA to the anticipated threat, the U.S. Army has developed modular BA that can be readily adjusted. These BA configurations range from no armor to heavy armor where the full set of soft armor, and ceramic front, back, and side plates are worn on the torso. The effectiveness of BA for protecting individuals is well-documented [2]. However, the tradeoff between the increased survival enabled by BA and the decreased performance associated with added mass has yet to be quantified. Body armor increases protection but also adds a thermal burden by impeding the dissipation of metabolic heat generated by physically active soldiers and increases metabolic rate from carrying additional mass from BA. Increased thermal burden adds to the risk of heat illness and effects human performance by distributing blood away from working muscles.

Heat illnesses typically result from a combination of three main elements, 1) environment (air temperature (T_a_), wind velocity (*V*), relative humidity (RH), and mean radiant temperature (T_mr_)), 2) metabolic heat (M) produced by the human, and 3) clothing biophysical properties (thermal insulation, vapor permeability, wind effects) [3]. With increases in thermal strain, not only does an individual become at higher risk of heat injury or illness, his or her work and endurance capacity decreases. This is especially important, as when working at higher intensities, maximal oxygen uptake (VO_2max_) decreases as thermal strain increases [4] and this decreased VO_2max_ increases work intensity [5]. Increases in work intensity and metabolic heat occur as a direct result of added human mass [6] or additional external load [7–8].

A number of studies have examined the effects of wearing BA on thermal strain and work capacity [9–11]. Cadarette et al. [9] studied soldiers wearing varied BA configurations working at moderate-intensity in a hot and dry environment, finding complexity in design features for mitigating thermal strain. Cadwell et al. [10] studied the effects of BA on individuals working at low-intensity in hot/humid conditions, finding increases in thermal and cardiovascular strain. Stewart and Hunt [11] found only minor heat strain effects on individuals wearing BA while operating armored vehicles within hot-humid conditions. Each of these studies examined the thermophysiological responses of the test volunteers but did not consider the biophysical properties or additional mass of the different BA configurations and clothing ensembles, or mathematically model the effects on thermoregulation. Using set mathematical methods to compare each unique ensemble in the context of the activity and environmental condition is an important step in evaluation of thermal performance of personal protective equipment [12].

The objectives of this effort were to quantify the biophysical properties of the various BA configurations, and mathematically predict the associated thermophysiological responses of the human working under hot/dry, hot/humid, and temperate environmental conditions. This study hypothesizes that using measured ensemble biophysics and thermoregulatory modeling and simulation of varied environments provides an ethical and cost efficient method of assessment, while avoiding complex human use evaluations.

## Materials and Methods

### Sweating thermal manikin and climate chamber

**Fig 1:**
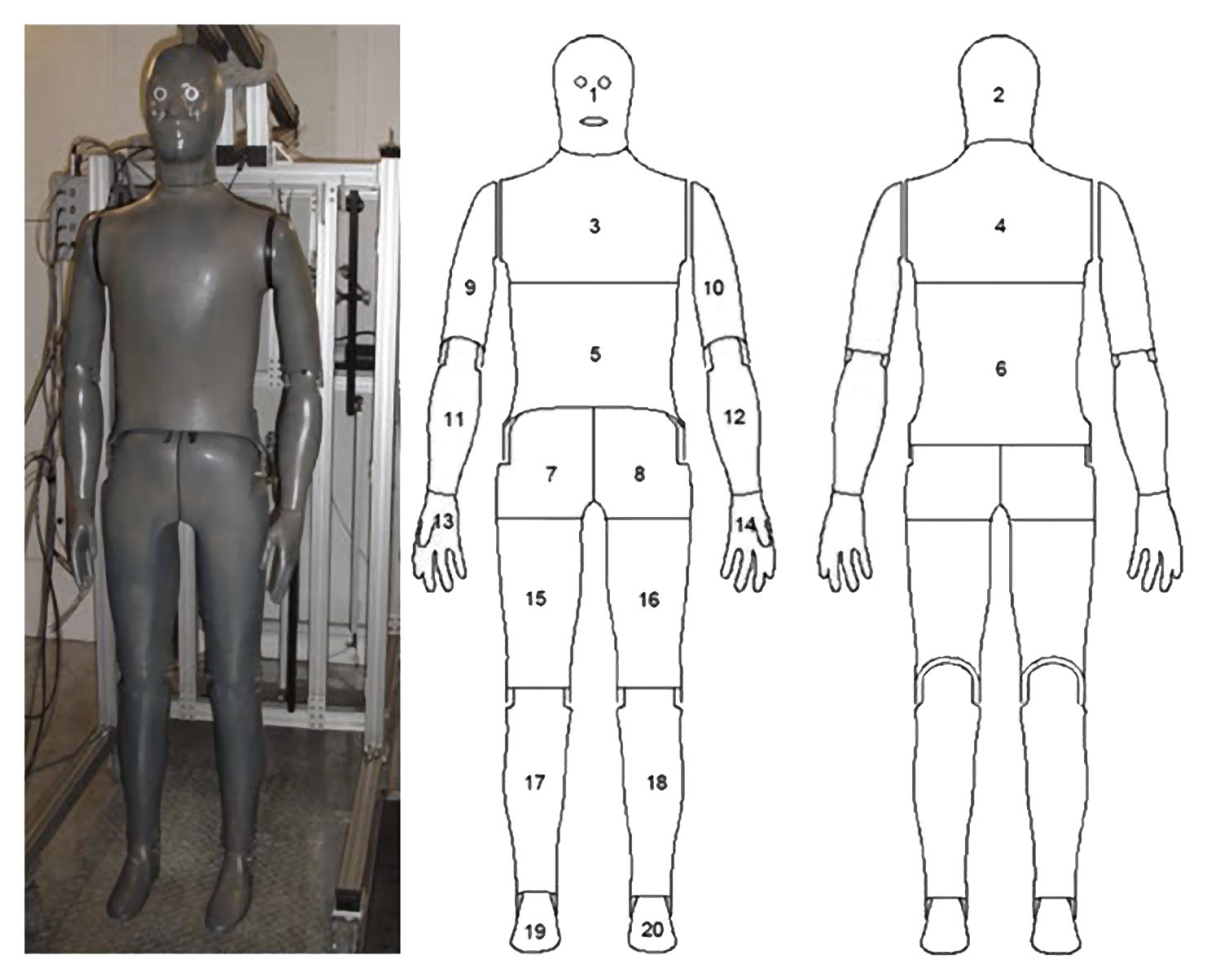
Twenty zone thermal sweating manikin (“Newton”, Measurement Technologies Northwest, Seattle, WA), operated in a climate controlled wind tunnel to derive thermal and evaporative resistances.

Biophysical tests were conducted using a thermal sweating manikin (Newton 20 zone, Measurement Technologies Northwest, Seattle, WA). The sweating heated manikin is comprised of 20 independent zones to simulate metabolic heat production and measure heat flux regionally (Fig 1). The manikin can also distribute water to the manikin surface to simulate sweating and enable measurements of evaporative heat loss. The manikin is located in a climate controlled wind tunnel at the U.S. Army Research Institute of Environmental Medicine (USARIEM) (Natick, MA).

### Body armor configurations

**Fig 2:**
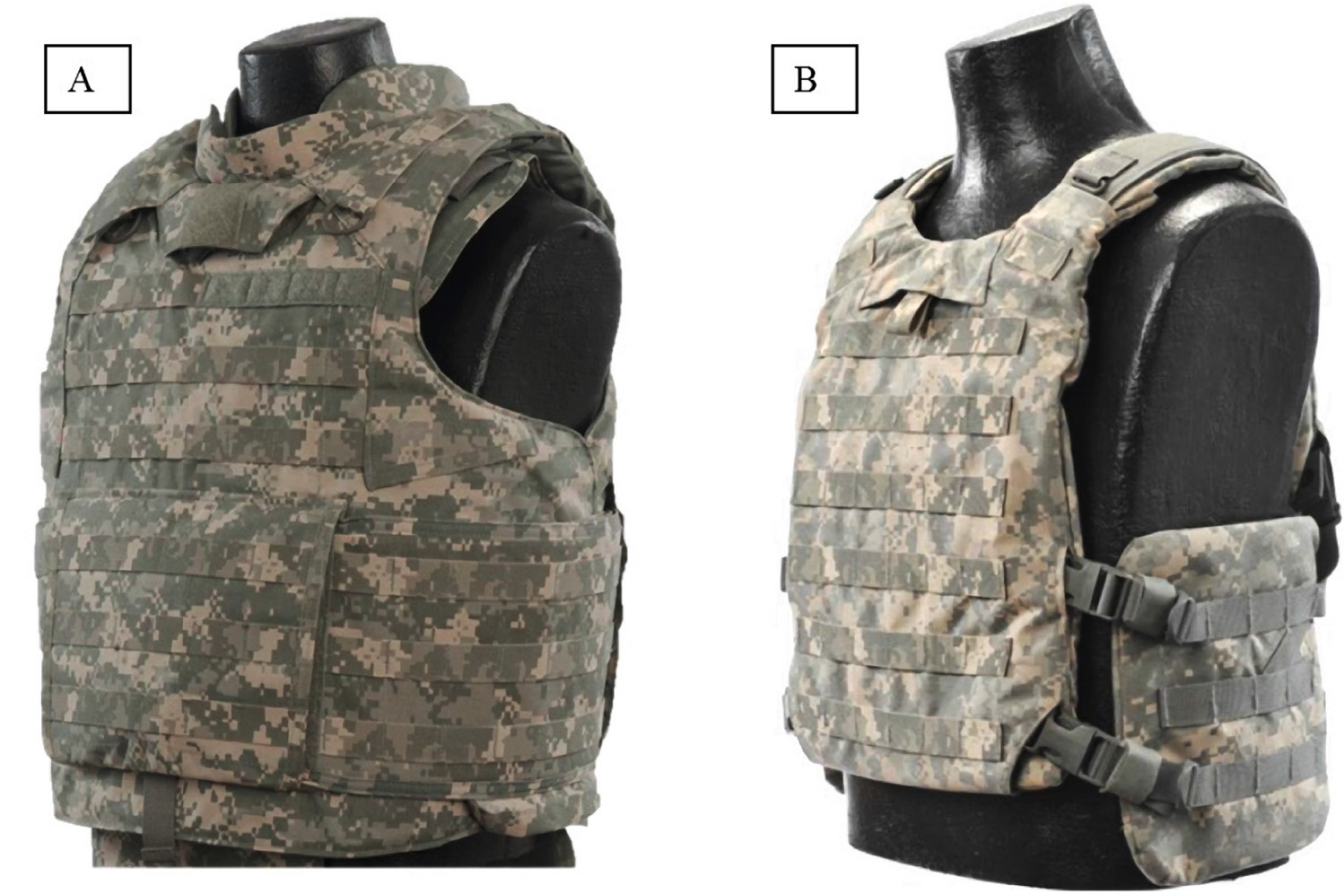
Soft armor vest and plate carrier used in configurations BA-1 through BA-7. Foot note: A—soft armor vest (IOTV) configurations (BA-1, BA-5, BA-6, and BA-7); B—plate carrier configurations (BA-2, BA-3, BA-4).

**Fig 3:**
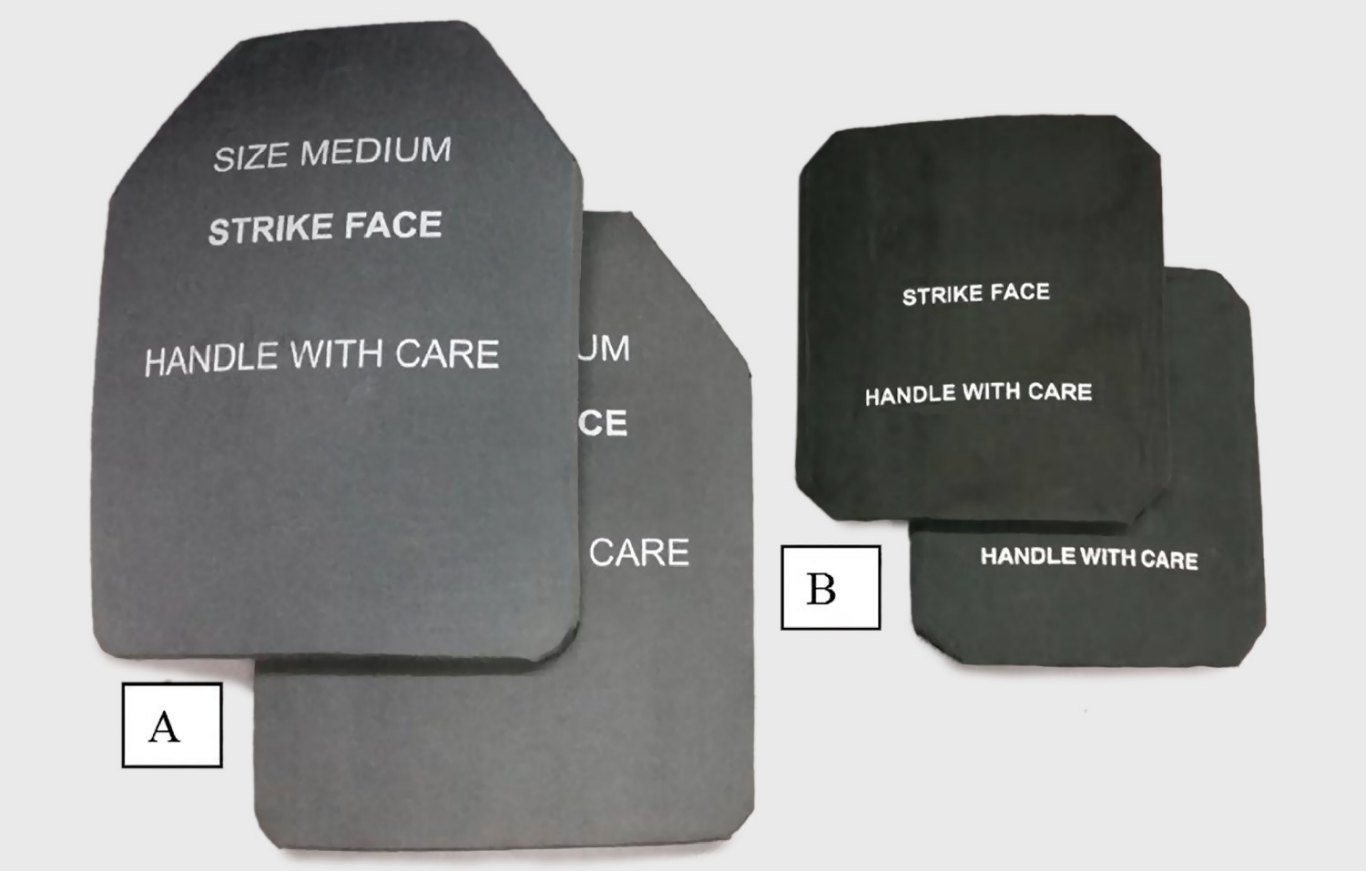
Ballistic plate inserts used in configurations BA-3 through BA-7. Foot note: A is front and back plates; B is side plates.

A total of eight configurations were tested; a baseline clothing ensemble with no BA and seven variants with increasing levels of ballistic protection added to the baseline configuration. A standard U.S. Army uniform was chosen for the baseline ensemble (BA-0): polypropylene boxer briefs, cotton socks, desert hot weather suede combat boots, eye protection (M frame; Oakley, Inc., Foothill Ranch, CA), Army Combat Shirt (ACS), Flame Resistant Army Combat Uniform (FRACU) pants, combat gloves (Max Grip; CamelBak Products, LLC, Petaluma, CA), and an Army combat helmet. Each of the ensemble configurations included this baseline clothing ensemble along with any added components of ballistic protection (BA-1 through BA-7) (Table 1) (Figs 2 and 3).

**Table 1:** Body armor configuration characteristics.

Abbreviation	Ballistic protection elements	Total Wt (kg)
**BA-0**	Baseline ensemble with no ballistic protection	2.5
**BA-1**	Baseline + Soft armor vest	5.2
**BA-2**	Baseline + plate carrier soft armor	7.3
**BA-3**	Plate carrier with front and back ballistic plates	10.7
**BA-4**	Plate carrier with front, back, and side ballistic plates	12.9
**BA-5**	Improved outer tactical vest (IOTV) with front and back ballistic plates	15.2
**BA-6**	IOTV with front, back, and side ballistic plates	17.0
**BA-7**	IOTV with front, back, side ballistic plates and groin and deltoid protection	17.9

### Biophysical assessments

Standard test methods were used to assess ensemble thermal resistance (R_ct_) and evaporative resistance (R_et_) (American Society for Testing and Materials (ASTM) F1291-10 and F2370-10) [13–14]. Following the assessments under ASTM standard conditions (0.4 m/s), additional tests were conducted at increased wind velocities (*V*) of approximately 1.2, and 2.0 m/s, to obtain coefficients representative of the wind impact on both R_ct_ and R_et_.

Thermal resistance (R_ct_) measures the transfer of heat from the thermal manikin, from convection and radiation, and is described by:

$$R_{ct}=\frac{\left(T_{s}-T_{a}\right)}{Q/A}[(m^{2}K)/W]$$ [Eq 1]

where *T*_*s*_ is the temperature of the manikin surface and *T*_*a*_ is the ambient temperature, both are in °C or K, *Q* is power input in Watts required to maintain the surface of the manikin at the set temperature of T_s_, and *A* is the area of the measurement surface in m^2^.

For R_ct_ testing to ASTM standard, the manikin surface (skin) temperature (T_s_) is set to 35°C and the ambient temperature (T_a_) set at 20°C, with the main requirement of having a 15°C temperature difference between the manikin and the environment. The relative humidity (RH) is maintained between 30–70%, ideally at 50%, and *V* is set to “still air” defined as 0.4 m/s (0.89 mph) [6]. With the exception of *V*, these same conditions are maintained and two additional tests are conducted at 1.2 and 2.0 m/s.

Evaporative resistance (R_et_) measures the heat lost from the manikin due to evaporation (i.e., simulated sweat), and in isothermal conditions (T_s_ ≈ T_a_), described by:

$$R_{et}=\frac{\left(P_{sat}-P_{a}\right)}{Q/A}\left[({\text{m}}^{2}\text{Pa}\right)/\text{W}]$$ [Eq 2]

where *P*_*sat*_ is vapor pressure in pascals at the surface of the manikin, which is assumed to be fully saturated, and *P*_*a*_ is vapor pressure, in pascals, of the ambient environment.

For R_et_ testing to ASTM standard, both the manikin T_s_ and the chamber T_a_ are set to 35°C, RH is set to 40%, *V* is set to 0.4 m/s, and the surface of the manikin is saturated with water [14]. When T_s_ and T_a_ are equal, measured heat loss is specifically due to evaporation. Similar to the R_ct_ tests, all conditions with the exception of *V*, are maintained and two additional tests are conducted at 1.2 and 2.0 m/s.

The additional tests conducted at increased *V* were used to create coefficients related to the *V* effect on thermal insulation and evaporative resistance [15].

Both R_ct_ and R_et_ are converted, respectively, to values of thermal insulation in units of (clo) and a vapor permeability index (i_m_). Measures of R_ct_ are converted into clo units; where 1 clo = 0.155 m^2^K/W or in a total ensemble (i.e., total insulation including boundary layers), the total insulation (I_T_) ∙ 6.45 = 1 clo [16–17]. Measures of R_et_ are converted into i_m_ units [18], a non-dimensional measure defined as:

$$i_{m}=\frac{60.6515\cdot \frac{P_{a}}{{}^{\circ}C}\cdot R_{ct}}{R_{et}}$$ [Eq 3]

Both clo and i_m_ are combined to establish an evaporative potential ratio (i_m_/clo) used to describe the ensemble’s insulation and evaporative performance potential in any environmental condition [19].

### Predictive modeling

Metabolic cost of walking ($\dot{M}$_w_) for a walking speed of 1.34 m/s (3 mph) on blacktop surface and level grade were estimated and adjusted for mass differences of each configuration (Table 1). Using an equation from Pandolf et al. [8], specific metabolic costs for each ensemble were estimated as:

$$M_{W}=1.5\cdot W+2.0\cdot \left(W+L\right)\cdot {\left(\frac{L}{W}\right)}^{2}+ŋ\cdot \left(W+L\right)\cdot \left(1.5\cdot V^{2}+0.35\cdot V\cdot G\right)$$ [Eq 4]

where *M*_*w*_ is metabolic cost of walking (or standing) (in watts); *W* is body mass (kg); *L* is load mass (kg); ŋ is terrain factor; *V* is velocity (m/s); *G* is slope or grade (%). The terrain factor category used was 1.0 is black top road or treadmill [8]. This equation, in contrast to most other equations, estimates metabolic cost of locomotion and accounts for addition of external load [20].

### Simulated environment and scenarios modeled

Modeling analyses assumed a healthy male, normally hydrated and heat acclimatized, with a body mass of 70 kg, and a height of 172 cm. Three environmental conditions (T_a_, %RH, *V*) were simulated: hot/dry (desert) (49°C, 20%, 1 m/s), hot/humid (jungle) (35°C, 75%, 1 m/s), and temperate (35°C, 50%, 1 m/s). Each environmental condition was simulated under 50% solar load conditions, where mean radiant temperature (T_mr_) is approximately T_a_ + 20 (i.e., desert = 69°C, jungle = 55°C, temperate = 55°C).

A modified version of an empirical mathematical method [21–22] was used to predict rise in core body temperature (T_c_) for each BA configuration that included solar loading effects [23]. This method predicts maximal continuous work times based on inputs of ensemble biophysical properties (clo, i_m_), environmental conditions (T_a_, RH, T_mr_, *V*), work rate, and human anthropometry (height, mass, surface area (m^2^)) and physiological status (hydration, heat acclimatization, and initial core and skin temperatures).

Specifically, an increase in T_c_ was estimated- using an empirically derived method from Givoni and Goldman [21] where:

$$T_{cf}=T_{c,0}+0.004\cdot M+0.0025+0.0011\cdot Dry+0.8\cdot \text{exp}(0.0047\cdot \left(E_{req}-E_{\text{max}}\right)$$ [Eq 5]

where *T*_*cf*_ is final core temperature, *T*_*c*,*0*_ is the initial core temperature, *M* is metabolic rate, *Dry* is radiative and convective heat transfer, *E*_*req*_ is evaporation required, and *E*_*max*_ is maximal evaporative capacity. Ensemble biophysical properties play a significant part in the rise of T_c_ by influencing E_req_ and E_max_ functions. The following heat exchange equations underpin the prediction of E_req_ and E_max_:

$$Dry=\frac{6.45}{I_{T}}\cdot A_{D}\cdot \left(T_{db}-{\bar{T}}_{sk}\right)$$ [Eq 6]

$$E_{req}=M-W_{ex}+Dry$$ [Eq 7]

$$E_{max}=LR\cdot 6.45\cdot \frac{i_{m}}{clo}A_{D}\cdot (P_{s,sk}-\left(\text{RH}\cdot P_{a}\right)$$ [Eq 8]

where *Dry* is the dry heat exchange, *A*_*D*_ is the surface area of the manikin/human (m^2^), *T*_*db*_ is the dry bulb temperature (°C), $\overset{-}{T}$_*sk*_ is the average surface skin temperature (°C), *W*_*ex*_ is the amount of external work performed, *LR* is Lewis relation (non-dimensional ratio), *P*_*s*,*sk*_ is saturated vapor pressure at the skin temperature (pascal), *P*_*a*_ air vapor pressure (pascal).

Two upper safety limits for T_c_ were assumed; one being a conservative value of 38.0°C [24], and the second 39.0°C value, being less conservative, aligned to military operational upper limits [25].

## Results

Table 2 shows estimated metabolic costs associated with walking at 1.34 m/s for each BA configuration, and the associated biophysical measures for each BA level. The $\dot{M}$_w_, and the associated heat production, increased with the increased mass as layers of ballistic protection are added from BA-1 to BA-7. It can also be seen that as BA protection levels increase, thermal and evaporative resistances increase as well.

**Table 2:** Metabolic cost estimated for walking at 1.34 m/s and biophysical measures at 0.4 m/s.

Configuration	$\dot{M}$_w_ (W)	Thermal Resistance (R_ct_) (clo)	Permeability Index (i_m_) (N.D.)	Evaporative Resistance (R_et_) (m^2^ Pa/W)
**BA-0**	300	1.37	0.449	28.7
**BA-1**	316	1.57	0.416	35.5
**BA-2**	323	1.59	0.398	37.5
**BA-3**	335	1.58	0.397	37.4
**BA-4**	343	1.57	0.392	37.6
**BA-5**	352	1.58	0.392	37.9
**BA-6**	360	1.58	0.370	40.1
**BA-7**	364	1.63	0.350	43.7

For modeling purposes, the effect of wind speed is a critical factor. Biophysical tests at multiple wind velocities were used to generate coefficient values specific to each ensemble’s clo and i_m_ changes with wind velocity. A power regression was used for each measure for both clo and i_m_ and these coefficients were used in the modeling process. This method establishes a constant value (*a*), representing the clothing property, the specific wind velocity (*V*), and the wind velocity coefficient (^g^) (Table 3).

**Table 3:** Wind velocity coefficient values (*V* ^*g*^) for each configuration.

Configuration	clo constant (*a*)	clo coefficient (^g^)	i_m_ constant (*a*)	i_m_ coefficient (^g^)
**BA-0**	1.09	-0.246	0.41	0.104
**BA-1**	1.23	-0.264	0.40	0.042
**BA-2**	1.25	-0.263	0.39	0.020
**BA-3**	1.24	-0.26	0.38	0.037
**BA-4**	1.25	-0.253	0.38	0.030
**BA-5**	1.25	-0.253	0.38	0.029
**BA-6**	1.26	-0.248	0.36	0.023
**BA-7**	1.28	-0.263	0.35	0.002

**Fig 4:**
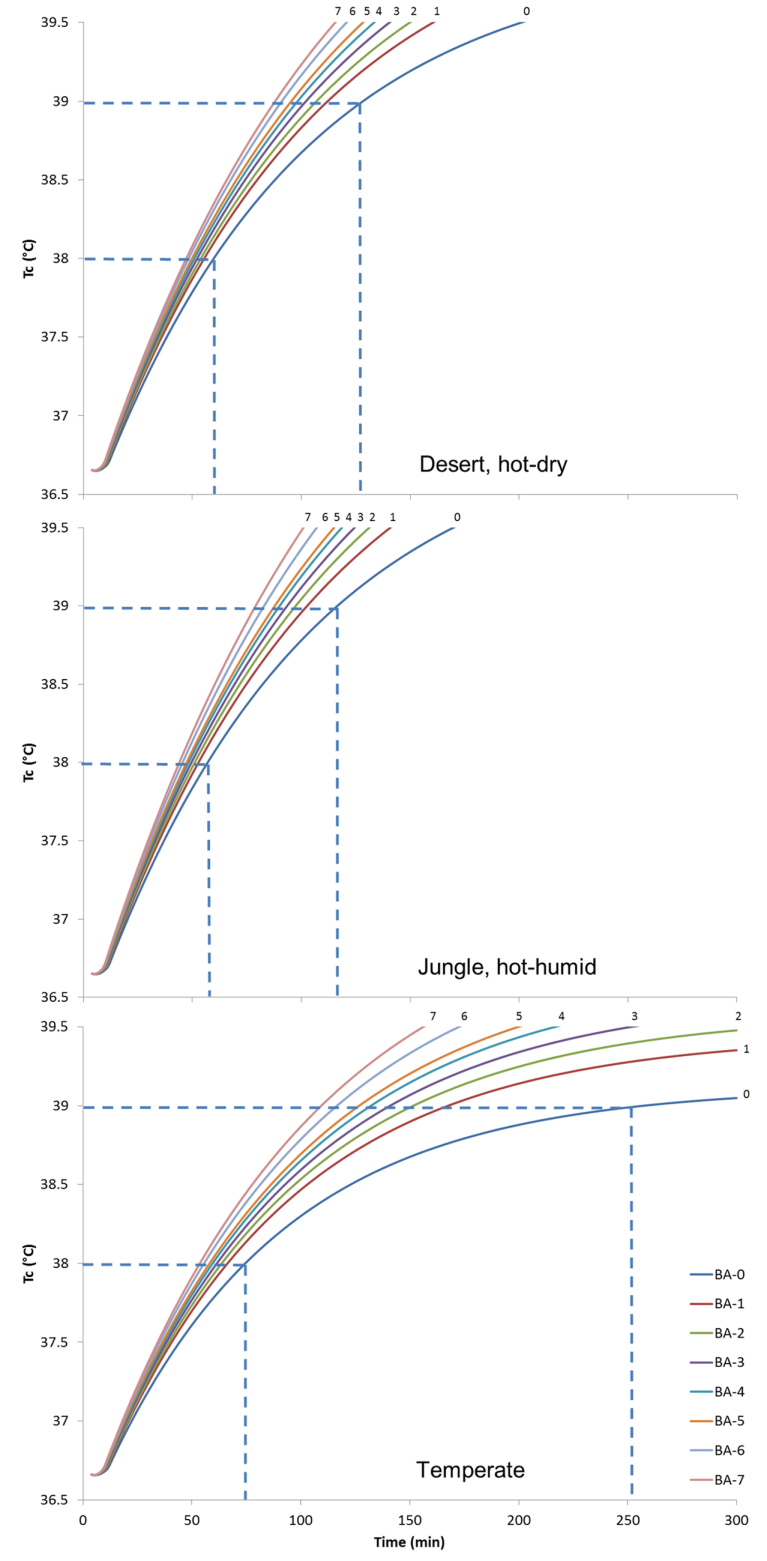
Predicted rise in T_c_ in desert, jungle, and temperate conditions. Foot note: Desert, hot-dry = 49°C, 20%, 1 m/s; Jungle, hot-humid = 35°C, 75%, 1 m/s; Temperate = 35°C, 50%, 1 m/s.

The estimated metabolic heat production and biophysical characteristics associated with each ensemble were used to predict the rise in T_c_ for three different environmental conditions (Fig 4). The predicted increases in T_c_ were used to identify maximal work times, i.e., the time before reaching industry levels (38.0°C) and military operation limits (39.0°C).

## Discussion

This study is the first that reports the biophysical properties, i.e., thermal and evaporative resistances, of current U.S. Army BA configurations. The study modeled increase in metabolic heat production associated with added mass of BA and predicted the effects of wearing each level of BA on human thermal responses. The increased insulation and vapor resistance associated with added ballistic protection added a thermal burden by reducing heat loss to the environment. The increased metabolic heat production associated with added ballistic protection added thermal burden as well. Our approach to quantify these tradeoffs will help material developers and combat infantry avoid using levels of BA that excessively compromise work performance. In response to combat injuries, overcompensation with increased protection is common, as can be seen with modern military’s insistence on blanket increases to ballistic protection.

Predicted results show that impact of BA on human thermal responses are dependent on environmental conditions. Predicted maximal work-times range from 64 to118 min in desert conditions, from 70 to 129 min in jungle conditions, and from 84 to 260 min in temperate conditions. It was observed from human studies that heat strain imposed by body armors were significant at 36°C, 60% RH [10], but were negligible at 31°C, 60% RH [26]. Modern dismounted military can readily tailor their BA protection levels based on expected threats and activities. The U.S. Marine Corps recognition of these types of thermal issues can be seen in changes from BA configurations used early in Iraq and Afghanistan [27–28] that incorporated more encapsulating and thermally burdensome soft armor vest to less encapsulating plate carrier with less soft armor. This shift is analogous to changing from BA-6 to BA-4 in the present study. As shown in Fig 4, this shift in increased endurance time from 90 min to 110 min at the temperate condition and does not change much between the other two conditions. The methods provided here offer a means of quantifying this tradeoff, enabling pragmatic adjustments of protection to meet survivability and human performance requirements of various missions.

This study evaluated the relationship of the entire ensemble’s biophysical characteristics to human thermal responses to environment and physical activity. The thermal and evaporative resistances reported in Table 2 are the whole body values. It is also important to note the effects of regional ensemble changes that may impact human thermoregulation (e.g., use of body armor plates). Being divided into 20 independent zones, the thermal manikin allows for some regional analysis, specific to items of clothing (e.g., torso and BA, head and helmets) [29]. These independent zones of the manikin can provide clo and i_m_ measures specific to each and each section can be accounted for by their relative surface area. In this study the configurations with added BA have significant reductions to the evaporative potential specific to the torso; where R_et_ at 0.4m/s ranged from 40.23 (BA-0; no armor) to 112.18 m^2^Pa/W (BA-7; full armor). These torso zones account for approximately 24% of the manikin total surface area (~0.44m^2^), chest (7%), shoulders (6%), stomach (7%), and back (5%) (zones 3–6, Fig 1). The modeling method takes into account an overall sweating rate, assuming an average across the entire surface area. With the exception of the head, these areas of the torso have been shown to have the highest sweat rates (g∙m^-2^, h^-1^) compared to the rest of the body [30]. However, given the nearly impermeable nature of the BA plates on the torso, an argument could be made that a significant local reduction of sweating efficiency could be seen with the inclusion of these components.

Metabolic heat production estimated by the Pandolf equation [8] only accounts for increase in energy cost due to carrying mass. BA-7 is about 15 kg heavier than BA-0, and this increases metabolic heat production by 64 W during walking at 1.34 m/s. Work from Dorman and Havenith [31] found that increases in energy costs while wearing protective clothing were not entirely associated with mass, suggesting there are significant ergonomic factors such as hobbling or unfavorable causes of increased energy demands. The modeling approach presented here does not address the ergonomic aspects of each of the BA configurations (e.g., form, fit), nor the potential biomechanical effects associated with wearing different BA configurations (e.g., hobbling, decreased range of motion) [32–33]. However, relatively simple adjustments to predictions could be made to represent increases in metabolic costs due to improper form or fit where assumed.

## Conclusions

This work quantified the biophysical characteristics of current U.S. Army BA configurations and mathematically predicted thermophysiologic effects of wearing each BA level when walking at 1.34 m/s in three different environmental conditions. Measured biophysical assessments and predictive modeling results show a quantifiable relationship exists between increased BA protection and mass, and an increased thermal burden and decreased work capacity.

Modeling and simulation methods such as those described in the present study should be incorporated as a foundational part of assessing tradeoffs between increased protection and human performance prior to human testing and use of various BA configurations. This modeling and simulation approach to simulating predicted heat strain and work performance is time- and cost- effective, and offers a quantitative way of improving mission planning and protective clothing development efforts, and minimizing the need for testing with human volunteers.

## Supporting Information

S1 File Measured thermal and evaporative resistances. (XLSX)
